# Iron amendment decreases methane emissions from subtropical paddies by altering soil microbial communities

**DOI:** 10.1128/spectrum.04000-25

**Published:** 2026-02-24

**Authors:** Zijian Qiu, Mingcheng Hu, Beibei Chen, Yaou Long, Keishi Senoo, Weishou Shen

**Affiliations:** 1 Jiangsu Key Laboratory of Atmospheric Environment Monitoring and Pollution Control, Collaborative Innovation Center of Atmospheric Environment and Equipment Technology, School of Environmental Science and Engineering, Nanjing University of Information Science and Technology 554690, Nanjing, China; 2 Department of Applied Biological Chemistry, Graduate School of Agricultural and Life Sciences, The University of Tokyo, Tokyo, Japan; 3 Collaborative Research Institute for Innovative Microbiology, The University of Tokyo, Tokyo, Japan; Connecticut Agricultural Experiment Station, New Haven, Connecticut, USA

**Keywords:** methane mitigation, nitrogen reduction, iron amendment, microbial community

## Abstract

Methane (CH_4_) emissions from flooded paddy fields, exacerbated by excessive nitrogen (N) fertilizer application, trigger serious climate challenges. The impact of reducing N fertilization rate combined with iron (Fe) amendment on CH_4_ emissions remains unclear. This 4-year field study (2020–2023) investigated the effects of 100%, 80%, 60%, and 0% of the conventional N (urea and commercial organic manure) fertilization rate (100%N, 80%N, 60%N, and 0%N) as well as 80%, 60%, and 0% of the conventional N with the Fe powder (≥99% purity) amendment (80%N + Fe, 60%N + Fe, and 0%N + Fe) on CH_4_ emissions from subtropical rice paddies. The results revealed that 60%N + Fe treatments decreased cumulative CH_4_ emissions by 43.79% compared to the non-amended treatment, and by 57.33% in relative to the 100%N treatment in the 2023 rice season (*P* < 0.05). Meanwhile, Fe amendment significantly lowered the *mcrA*/*pmoA* ratio, which facilitated the decrease in CH_4_ emissions. Community assembly analysis showed that Fe amendment enhanced stochastic processes in methanogens at 60% of conventional N but reduced dispersal at 80% of conventional N, with opposite trends for methanotrophs. Co-occurrence networks demonstrated increased connectivity and reduced modularity under Fe amendment. Moreover, soil Fe^2+^ content and methanogen community structure, as critical drivers, were negatively correlated with CH_4_ flux and cumulative emissions (*P* < 0.05). Taken together, Fe amendment is a potent strategy to mitigate CH_4_ emissions under reduced N fertilization, offering a green production solution for global paddy systems.

**IMPORTANCE:**

This study clarified the effects of Fe amendment on CH_4_ emissions from subtropical paddy fields under various N fertilization rates through a 4-year *in situ* field experiment. We found that the Fe amendment combined with reduced N fertilization rates decreased CH_4_ emissions, in particular under the 60% of conventional N fertilization rate. Furthermore, the Fe amendment lowered the *mcrA*/*pmoA* ratio. Moreover, the Fe amendment increased connectivity while reducing modularity in co-occurrence networks of methanogen communities. Soil Fe^2+^ content and methanogen community structure were key drivers of CH_4_ emissions. The findings provide an insight into the microbial mechanisms of mitigating CH_4_ emission from flooded paddy soils through the Fe amendment.

## INTRODUCTION

Methane (CH_4_), as a potent greenhouse gas whose greenhouse effect is second only to that of carbon dioxide, has exacerbated global climate warming (1). Flooded paddy fields are considered to be one of the important sources of atmospheric CH_4_. Approximately 90% of rice cultivation globally occurs in flooded environments, which greatly promotes the generation of CH_4_ (2). Asian countries emit the world’s largest CH_4_ from rice paddies, in which China’s CH_4_ emissions from paddy fields in recent years are estimated to be between 6.21 Tg yr^−1^ and 6.57 Tg yr^−1^ (3). However, there are obvious differences in CH_4_ emissions from paddy fields in different regions. For instance, the average CH_4_ emissions from paddy fields in inland China during the rice growing season are higher than those from coastal paddy fields (3, 4). Such differences in emissions are closely related to various factors such as soil properties, climatic conditions, and agricultural management measures (3).

In paddy fields, methanogens are a group of microorganisms that can convert simple organic compounds into CH_4_ under anaerobic conditions. They utilize organic carbon sources in the soil, such as plant residues and root exudates, to produce CH_4_ through a series of complex metabolic processes (5). The methanogens in paddy fields mainly include Methanomicrobiales, Methanosarcinales, Methanocellales, and Methanobacteriales (6). Methanotrophs can use CH_4_ as a carbon source and energy source for growth and metabolism, thereby decreasing CH_4_ emissions into the atmosphere (7, 8). The methanotrophs are mainly divided into Type I (γ-Proteobacteria) and Type II (α-Proteobacteria) (9). Type I methanotrophs usually dominate in paddy fields, such as the genus *Methylococcus* (6). The methyl coenzyme M reductase (*mcr*) of CH_4_ metabolizing archaea catalyzes the reversible reaction between CH_4_ and methyl coenzyme M, while the particulate methane monooxygenase (*pmo*) of CH_4_ oxidizing bacteria is responsible for oxidizing CH_4_ to methanol. The *mcrA* and *pmoA* genes encoding these two enzymes are usually used as CH_4_ metabolism marker genes, and their abundance can reflect the abundance of methanogens and methanotrophs in the environment, respectively (10).

Excessive application of synthetic N fertilizers can lead to a series of environmental problems, such as ammonia volatilization, nitrous oxide emissions, non-point source pollution, and groundwater hydrochloride pollution (11, 12). Hence, it is urgent to reduce the application of synthetic N fertilizers. Several action plans have been promoted by the Chinese government for reducing synthetic fertilizer use and improving the use efficiency. The impact of N fertilizers on CH_4_ emissions from paddy fields is complex, and the current research has no consensus yet. Some studies have shown that the application of N fertilizers such as urea will increase CH_4_ emissions in paddy fields, and these may be owing to N fertilizers promoting rice growth, providing more carbon substrates, changing the redox state of the soil and water, and meanwhile, the ammonium N produced by hydrolysis inhibiting the activity of methanotrophs (6, 13, 14). Moreover, with the increase in the application rate of N fertilizers, the CH_4_ flux from paddy fields increases significantly (1). Reducing the N fertilizer application rate from 250 kg ha^−1^ to 150 kg ha^−1^ significantly decreased the CH_4_ emission intensity from paddy fields, which may be owing to decreasing the available N in the soil and the activity of microorganisms, thus reducing the generation of CH_4_ (15). However, other studies have also reported that the N fertilizer application can decrease CH_4_ emissions (16–18). At high nitrogen input levels, the activity and population of methanotrophs increase significantly, leading to more CH_4_ being oxidized before emission (18). Meanwhile, reducing the N input may also restrict the growth of rice and the release of root exudates and indirectly affect CH_4_ emissions (6). Overall, in climate change scenarios, reducing the application of N fertilizers is considered a potential strategy to mitigate CH_4_ emissions from paddy fields.

Although reducing N fertilizer application can alleviate the negative environmental impacts of N in paddy fields (19), long-term decrease in application may lead to reduced rice yield. Applying iron (Fe) powder can enhance biological N fixation in paddy soil, improve N fertility levels, and maintain stable production (19–21). The application of fertilizers containing iron oxides and Fe^3+^ can also effectively decrease CH_4_ emissions from paddy fields (22, 23). Fe can act as electron acceptors and participate in redox reactions in the soil, inhibiting the growth and activity of methanogens, thereby decreasing the generation of CH_4_ (24, 25). After applying medium and high rates of Fe in paddy fields, seasonal CH_4_ emissions were significantly decreased by 65% and 62%, respectively (26). The Fe amendment not only influenced CH_4_ emissions but also altered the abundance and community structure of methanogens and methanotrophs. For instance, the copy number and potential activity of methanogens were significantly decreased, while methanotrophs showed the opposite trend, and their community tended to shift toward being dominated by Type II methanotrophs (26). Therefore, the Fe amendment can also change the processes of CH_4_ generation and oxidation by regulating the microbial community structure.

Currently, the information about the impacts of the Fe amendment—especially following China’s efforts to decrease synthetic fertilizer use and improve their efficiency—on CH_4_ emissions, methanogens, and methanotrophs was rather limited. Hence, a 4-year *in situ* field experiment of different N fertilization rates in combination with Fe amendment was conducted. This study aims to clarify (i) the impact of reducing N fertilization rate and Fe amendment on CH_4_ emissions, (ii) the responses of methanogens and methanotrophs in the soil, and (iii) the key microbial taxa and soil physicochemical properties driving CH_4_ emissions. This study may provide a novel approach for mitigating CH_4_ emissions from subtropical paddy fields.

## MATERIALS AND METHODS

### Site description

The field site is situated in Liuhe District, Nanjing City, Jiangsu Province, China (32°21′N, 118°51′E), where rice-wheat rotation is a dominant farming system. The climate of the area is subtropical monsoon with a mean annual precipitation of 941.6 mm, a mean annual temperature of 15.6°C, and an annual frost-free period of around 254 days. The soil is a stagnic anthrosol. Basic soil properties of the cultivated layer (0–20 cm depth) include the following: pH (H_2_O) 5.9, total N (TN) 1.68 g kg^−1^, organic matter 25.4 g kg^−1^, available phosphorus 32 mg kg^−1^, and available potassium 108 mg kg^−1^.

### Experiment design

A 4-year field trial (2020–2023) was conducted using 28 experimental plots (4 m × 5 m each) planted with the rice cultivar Ningjing 8 (*Oryza sativa* L. subsp. *japonica*), cultivated annually from June to November. The treatments included 100%, 80%, 60%, and 0% of the conventional N fertilization rate (100%N, 80%N, 60%N, and 0%N) and 80%, 60%, and 0% of the conventional N fertilization rate combined with Fe amendment (80%N + Fe, 60%N + Fe, and 0%N + Fe). All treatments had four replicates and were designed as randomized blocks. The conventional N fertilizer application rate was 315 kg N hm^−2^ (including urea N and manure N), and the N fertilizers were applied to the field for the basal fertilizer, first supplementary fertilizer, and second supplementary fertilizer, with a ratio of 6:3:1. The application rates of manure, calcium-magnesium phosphate, and potassium chloride were 70 kg N hm^−2^, 60 kg P_2_O_5_ hm^−2^, and 105 kg K_2_O hm^−2^, respectively, which were also applied as basal fertilizers in addition to the urea. Prior to the initiation of waterlogging in May 2019, the Fe powder (zero-valent iron, >99% purity, Shijiazhuang, China) was applied to the soil surface at 5,000 kg hm^−2^ and left to oxidize. Sampling was not performed during the 2019 rice season to allow the applied Fe powder to convert into available Fe. Detailed fertilization and field management have been published previously (19).

### CH_4_ emission measurement

A closed chamber method evaluated the CH_4_ flux from each field plot. The CH_4_ flux was measured every 3–5 days if higher than the background flux rate and every 7–10 days if closer to the background flux rate throughout the rice season. In brief, each chamber base was tightly closed by a chamber box (0.5 m × 0.5 m × 0.6–1.2 m) with its height adjusted from 0.6 m to 1.2 m depending on the heights of the plants. A gas sample was collected into a 15 mL vial every 15 min for a total period of 30 min (i.e., 0, 15, and 30 min); the temperature inside the chamber was also measured at each sampling. Soil moisture and temperature at a 10 cm depth were measured every hour with the Decagon 5TM volumetric water content and temperature sensor and recorded by a Meter ZL6 Advanced Cloud Data Logger (Meter Group, Inc., Pullman, Washington, DC, USA). The concentration of CH_4_ was determined by a gas chromatograph with a flame ionization detector (Agilent 7890B, Wilmington, DE, USA). CH_4_ fluxes and cumulative CH_4_ emissions were calculated using equations 1 and 2, respectively (27):


(1) F=ρ×H×(Δc/Δt)×273/(273+T)

where *F* is the CH_4_ (mg m^−2^ h^−1^) flux, *ρ* is the gas density of CH_4_ (0.717 mg cm^−3^) under standard conditions (0°C, 101 kPa), *H* is the height of the chamber above the water layer (m), Δ*t* is the sampling interval time (h), Δ*c* is the concentration change of CH_4_ (mg m^−3^) within Δ*t*, Δ*c*/Δ*t* is the cumulative emission rate of CH_4_ (mg m^−3^ h^−1^) in the chamber, and *T* is the mean temperature inside the chamber at each sampling (°C).


(2) $$S=\sum_{i=1}^{n}(F_{i}+F_{i+1})/2\times (t_{i+1}-t_{i})\times 24$$

where *i* is the *i*th sampling, *n* is the total number of sampling times, *F_i_* is the CH_4_ (mg m^−2^ h^−1^) emission flux for the *i*th sampling, and (*t_i+1_* − *t_i_*) is the interval of days between the *i*th and (*i +* 1)th sampling.

Additionally, the average CH_4_ flux was calculated by dividing the cumulative emissions by the number of sampling times.

### Soil physicochemical property measurement

Soil samples were collected during critical rice growth stages including seedling (the initial phase from seed germination), tillering (the period of lateral shoot emergence determining productive stem number), jointing (the transitional phase marked by basal internode elongation and panicle primordium differentiation), heading (the stage of panicle exsertion and anthesis), grain filling (the period of assimilate translocation to developing caryopses), and maturity (the final phase with grain ripening and dry weight stabilization) stages in both 2022 and 2023 growing seasons. Surface soils (0–20 cm) collected near the rice roots using soil drills were encapsulated in plastic bags. Fresh soil samples were first mixed well after passing through a 2 mm sieve after the removal of debris. Part of these mixed soil samples was stored in a 15 mL sterile centrifuge tube that was kept with dry ice and transported to the laboratory, and then immediately stored at −80°C. The remaining soil samples were stored at 4°C until they were required for the determination of physicochemical properties. The soil stored at −80°C was used to assess gene abundance and amplicon sequencing. The soil pH, electrical conductivity (EC), soil moisture content, total carbon, TN, nitrate N (NO_3_^−^–N), ammonium N (NH_4_^+^–N), Fe^2+^, and Fe^3+^ in the soil samples were measured according to reference 28.

### Real-time quantitative PCR analysis

Approximately 0.5 g of fresh soil stored at −80°C was used to extract total soil DNA using a DNA kit (HiPure Soil DNA Mini Kit, Magen, China) and detected by 1% agarose gel electrophoresis according to the instruction manual. The gene copy numbers of 16S rDNA, *mcrA*, and *pmoA* were sequentially detected using real-time fluorescence quantitative PCR (qPCR) technology. The amplification of qPCR was performed using a real-time fluorescence quantitative PCR instrument (CFX96; Bio-Rad, USA). Real-time fluorescence qPCR amplification was performed using a standard plasmid with a concentration gradient of 10^2^–10^6^ as a template for the standard curve. The qPCR reaction volume totaled 25 μL, including 5 μL of DNA template (DNA was diluted and mixed with double-distilled water and EASY Dilution in proportion before addition), 12.5 μL of TB Green Premix Ex Taq II, forward/reverse primer each 1 μL (10 μM), and 5.5 μL of sterilized double-distilled water. Sterilized double-distilled water was added to the negative control instead of the template DNA sample. The primer sequences and reaction program settings for the different genes involved in the reaction are listed in Table S1. The amplification efficiency of all the experimental results was >90%, and the dissolution curve showed a single peak.

### Sequence analysis

To avoid short-term disturbances on the microbial community, soil DNA samples from the 2022–2023 rice season were selected for high-throughput sequencing. The *mcrA* gene was amplified with the primer pair MLf (5′-GGTGGTGTMGGATTCACACARTAYGCWACAGC-3′) and MLr (5′-TTCATTGCRTAGTTWGGRTAGTT-3′). The *pmoA* gene was amplified with the primer pair A189 (5′-GGNGACTGGGACTTCTGG-3′) and A650 (5′-ACGTCCTTACCGAAGGT-3′). The purified amplified product (amplicon) connected to the sequencing adapter was used to construct a sequencing library, which was sequenced using the Illumina MiSeq-PE250 platform. Raw paired-end reads were assigned to each sample based on unique barcodes. A standard pipeline was used for quality control and clustering using the DADA2 plug-in unit in QIIME2 (https://qiime2.org/) to obtain amplicon sequence variants (ASVs), which were defined based on the exact sequence variants that differed by a single nucleotide. Briefly, after importing to QIIME2 with the manifest file, the sequences were demultiplexed using the q2-dmux plug-in. The demultiplexed sequences were then denoised and quality-filtered using the q2-dada2 plug-in according to the standard DADA2 protocol, and an ASV table and representative sequences were generated. Chimeric sequences and singleton ASVs (i.e., ASVs with a total number of sequences of only one in the whole sample) were excluded, and the sequences were annotated based on GraftM. Sequence data were uploaded to the NCBI SRA under the bioproject number PRJNA1293873.

### Neutral community model

A neutral community model was used to determine the potential importance of stochastic processes on community assembly (29). *N*_*m*_ is an estimate of dispersal between communities in this model. The parameter *N*_*m*_ determines the correlation between occurrence frequency and regional relative abundance, with *N* describing the metacommunity size and *m* being the immigration rate. The parameter *R*^2^ represents the overall fit to the neutral model. Calculation of 95% confidence intervals around all fitting statistics was done by bootstrapping with 1,000 bootstrap replicates.

### SPEC-OCCU plots

The ASV with both specificity and occupancy ≥ 0.7 was designated as a specialist. Specificity is defined as the mean abundance of species (S) in the samples of habitat (H), and occupancy is defined as the relative frequency of occurrence of S in the samples of H. The specific calculation formula was according to reference 30.

### Statistical analyses

Tukey’s tests, as part of one-way and two-way ANOVA, were performed using GraphPad Prism 10 software to analyze data on the CH_4_ emissions, gene abundance, and α-diversity. The DESeq2 package was used to establish the Manhattan plot. The corrplot package was used to establish the correlation heatmap. The rfPermute package was used to analyze data using the random forest variable significance test. The co-occurrence networks were visualized through Gephi 0.10.1 software.

## RESULTS

### CH_4_ emissions

**Fig 1:**
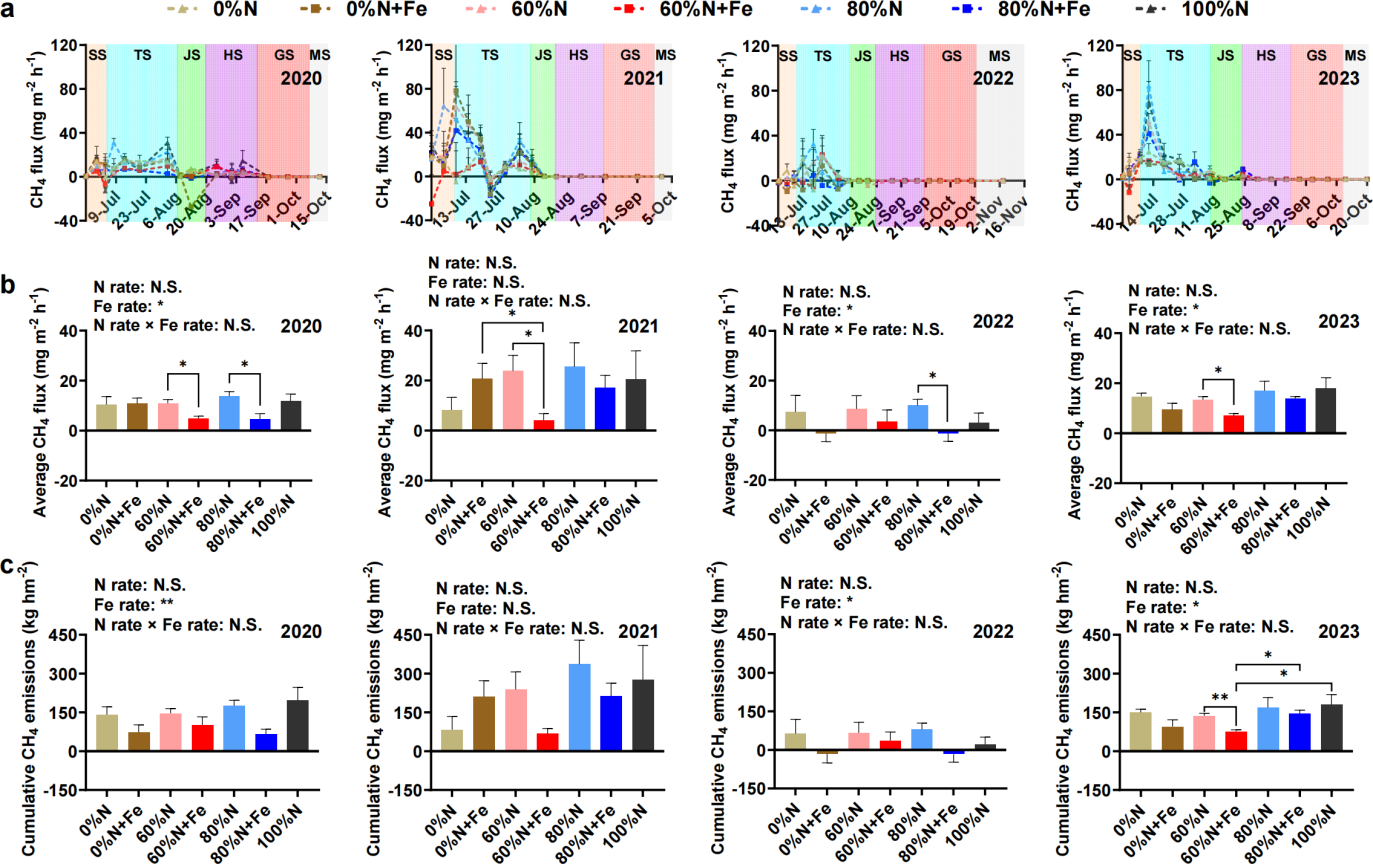
The CH_4_ fluxes (**a**), average CH_4_ fluxes during pulse peak period (**b**), and cumulative CH_4_ emissions (**c**) in rice paddy fields. Statistical analysis was conducted using one-way and two-way ANOVA. Significant differences between treatments were tested by Tukey’s HSD. SS, seeding stage; TS, tillering stage; JS, jointing stage; HS, heading stage; GS, grain filling stage; MS, maturity stage; N.S., not significant; *, *P* < 0.05; **, *P* < 0.01; ***, *P* < 0.001 (same below).

A pulse emission peak of CH_4_ was observed during the flooding period in the early rice season, especially at the tillering stage (Fig. 1a). Notably, higher emission peaks were detected around 2 weeks after basal fertilizer application in the 2021, 2022, and 2023 rice seasons, respectively, relative to the corresponding periods in 2020 (Fig. 1a). Soil moisture during the tillering stage generally remained higher than that in later growth stages (Fig. S1).

During the peak CH_4_ emission period (approximately 40 days after rice transplanting), Fe amendment significantly influenced average flux in 2020, 2022, and 2023 rice seasons (*P* < 0.05) (Fig. 1b). In 2020, the 60%N + Fe and 80%N + Fe treatment decreased average CH_4_ flux by 55.21% and 66.20% compared to equivalent N but non-Fe-amended treatments (*P* < 0.05). In 2021, the 60%N + Fe treatment achieved 82.85% and 80.34% reductions compared to 60%N and 0%N + Fe treatments, respectively (*P* < 0.05). In 2022, due to the disturbance of soil moisture conditions caused by high temperature and drought (Fig. S1), the 80%N + Fe treatment decreased from 9.95 mg m^−2^ h^−1^ in the 80%N treatment to −1.31 mg m^−2^ h^−1^ (*P* < 0.05). Notably, the 60%N + Fe treatment also had a 47.23% reduction compared to 60%N treatment in 2023 (*P* < 0.05) (Fig. 1b).

Fe amendment exerted significant impacts on cumulative CH_4_ emissions (Fig. 1c). In the 2020, 2022, and 2023 rice seasons, two-way ANOVA revealed that Fe amendment, rather than N fertilization rates, profoundly influenced cumulative CH_4_ emissions (*P* < 0.05). Specifically, according to one-way ANOVA, during the 2023 rice season, the 60%N + Fe treatment had a 43.79% reduction (*P* < 0.05) relative to its 60%N counterpart, while also showing significant decreases by 47.17% and 57.33% (*P* < 0.05) compared to 80%N + Fe and 100%N treatments, respectively (Fig. 1c).

### Real-time quantitative PCR assays

The Fe amendment did not exert statistically significant effects on either absolute or relative abundances of the *mcrA* gene at the tillering and jointing stages across four rice seasons. A notable exception occurred at the 2022 tillering stage, where the interactive effect of N fertilization rates and Fe amendment significantly influenced the absolute abundance of the *mcrA* gene (*P* < 0.05, Fig. S2). Additionally, N application rates alone showed a significant impact on the relative abundance of the *mcrA* gene during the 2023 jointing stage (*P* < 0.05, Fig. S2).

Similarly, the Fe amendment exhibited no statistically significant effects on absolute or relative abundances of the *pmoA* gene across the same observation periods. However, at the 2023 jointing stage, marked downregulation of *pmoA* gene absolute abundance was observed in both 0%N (7.90 × 10^5^ copies g^−1^ dry soil) and 60%N treatments (8.86 × 10^5^ copies g^−1^ dry soil) compared to the 100%N treatment (5.08 × 10^6^ copies g^−1^ dry soil) (*P* < 0.05, Fig. S3).

**Fig 2:**
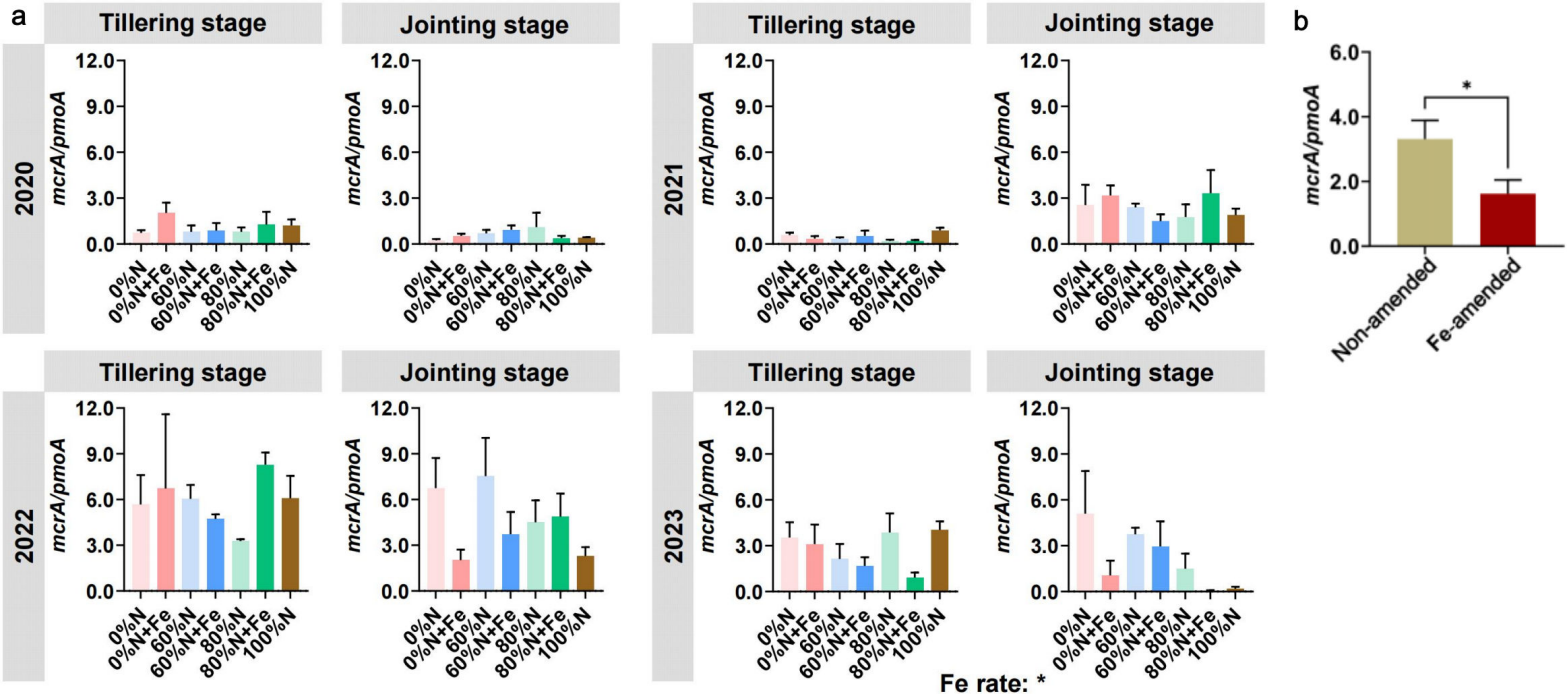
The ratio of *mcrA*/*pmoA* at the tillering and jointing stage (**a**). Statistical analysis was conducted using one-way and two-way ANOVA. Significant differences between treatments were tested by Tukey’s HSD. The ratio of *mcrA*/*pmoA* in Fe-amended and non-amended treatments during the 2023 rice season (**b**). Significant differences between treatments were tested by *t*-test.

No significant differences were found between the ratio of *mcrA*/*pmoA* in each treatment during the single growth stage, but the Fe rate had a significant effect on the whole in 2023 (Fig. 2a). Overall, the Fe amendment significantly reduced the ratio of *mcrA*/*pmoA* compared with no Fe amendment, decreasing from 3.31 to 1.62 (Fig. 2b).

### The methanogen and methanotroph community composition, diversity, and community assembly

**Fig 3:**
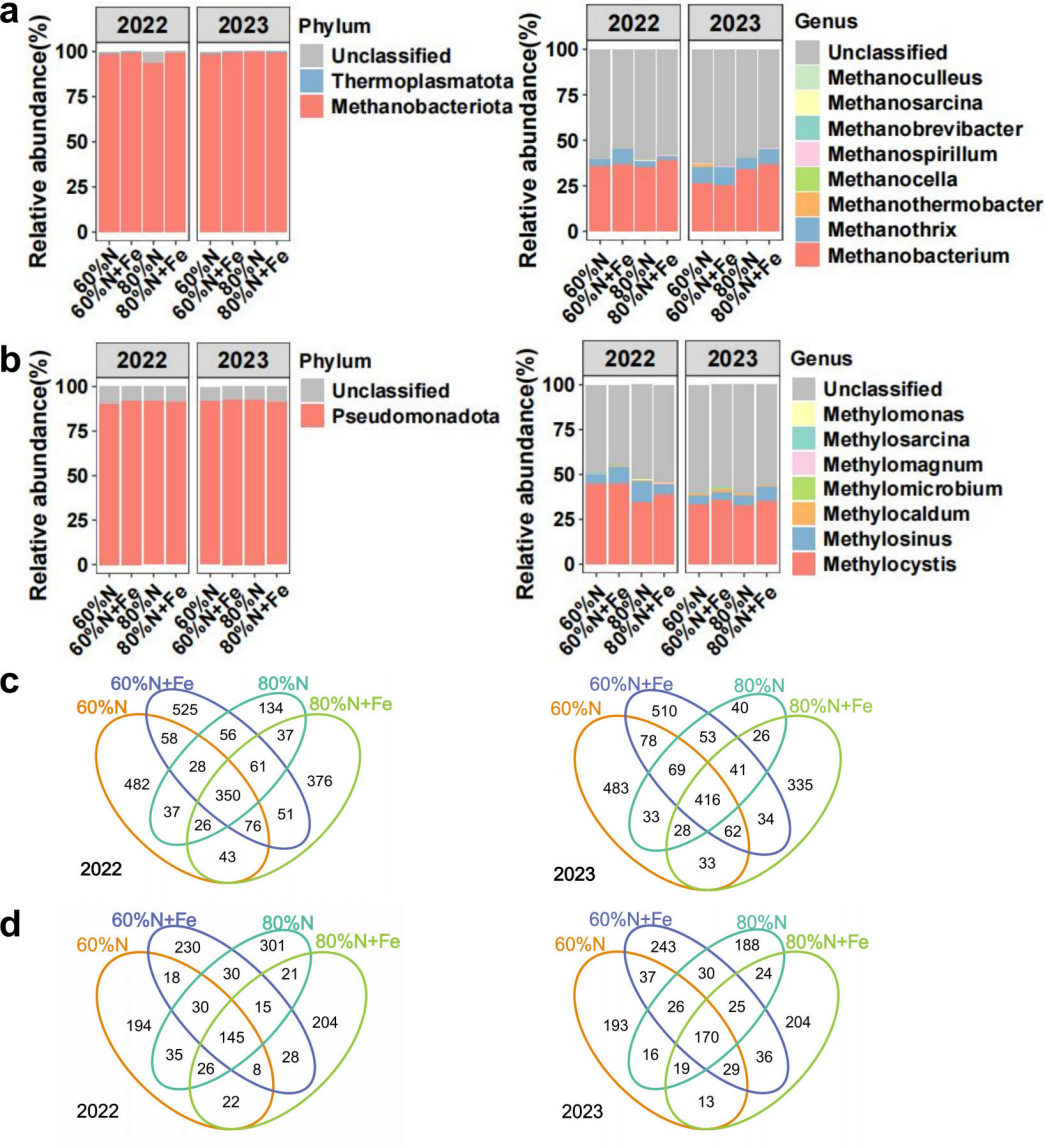
The relative abundance of methanogens (**a**) and methanotrophs (**b**) at the phylum and genus levels. The Venn diagram of the methanogen (**c**) and methanotroph community composition (**d**).

During 2022 and 2023 rice seasons, methanogen communities at the phylum level were primarily composed of Methanobacteriota (93.57%–99.52%), Thermoplasmatota (0.20%–1.18%), and unclassified taxa (0.14%–6.08%) (Fig. 3a). At the genus level, the main taxa were *Methanobacterium*, *Methanothrix*, *Methanothermobacter*, *Methanocella*, *Methanospirillum*, *Methanobrevibacter*, *Methanosarcina*, and *Methanoculleus*, totally accounting for 35.83%–45.79% (Fig. 3a). In addition, the 60%N + Fe treatment significantly increased the relative abundance of *Methanothrix* compared to the 80%N + Fe treatment (*P* < 0.05). Except for unclassified taxa, the methanotroph communities were primarily Pseudomonadota, with a relative abundance of 90.17%–92.44% (Fig. 3b). At the genus level, the main taxa were *Methylocystis*, *Methylosinus*, *Methylocaldum*, *Methylomicrobium*, and *Methylomagnum*, totally accounting for 39.72%–55.02% (Fig. 3b).

**Fig 4:**
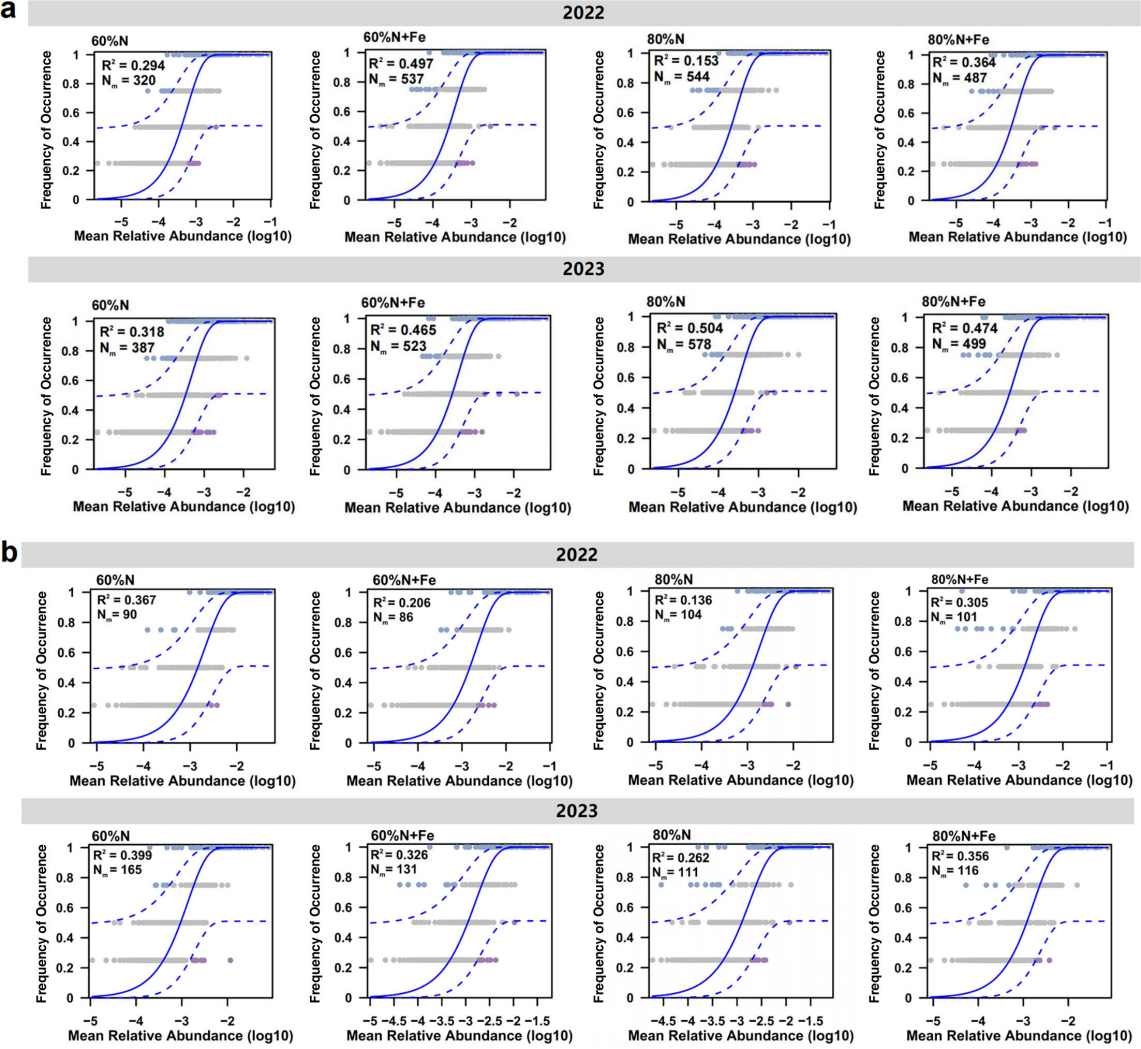
The methanogen (**a**) and methanotroph (**b**) community assembly based on neutral theory.

Methanogen communities exhibited fewer core operational taxonomic units (ASVs) across four treatments in the 2022 rice season (*n* = 350) compared to the 2023 rice season (*n* = 416) (Fig. 3c). Similarly, methanotroph communities showed fewer core ASVs in 2022 (*n* = 145) versus 2023 (*n* = 170) (Fig. 3d). No significant differences were detected in α-diversity or β-diversity (Bray-Curtis dissimilarity) between seasons for either community (Fig. S4). Combining the results of the two seasons, in the process of methanogen community assembly, the Fe amendment enhanced the influence of stochastic processes at the 60% of conventional N rate. The Fe amendment promoted species dispersal at the 60% of conventional N rate, while reduced species dispersal at the 80% of conventional N rate (Fig. 4a). In the process of methanotroph community assembly, the Fe amendment weakened the influence of stochastic processes at the 60% of conventional N rate, while enhancing the influence of stochastic processes at the 80% of conventional N rate. The Fe amendment reduced species dispersal at 60% of conventional N rate (Fig. 4b).

### Differential abundance taxa, specialists, and co-occurrence networks

**Fig 5:**
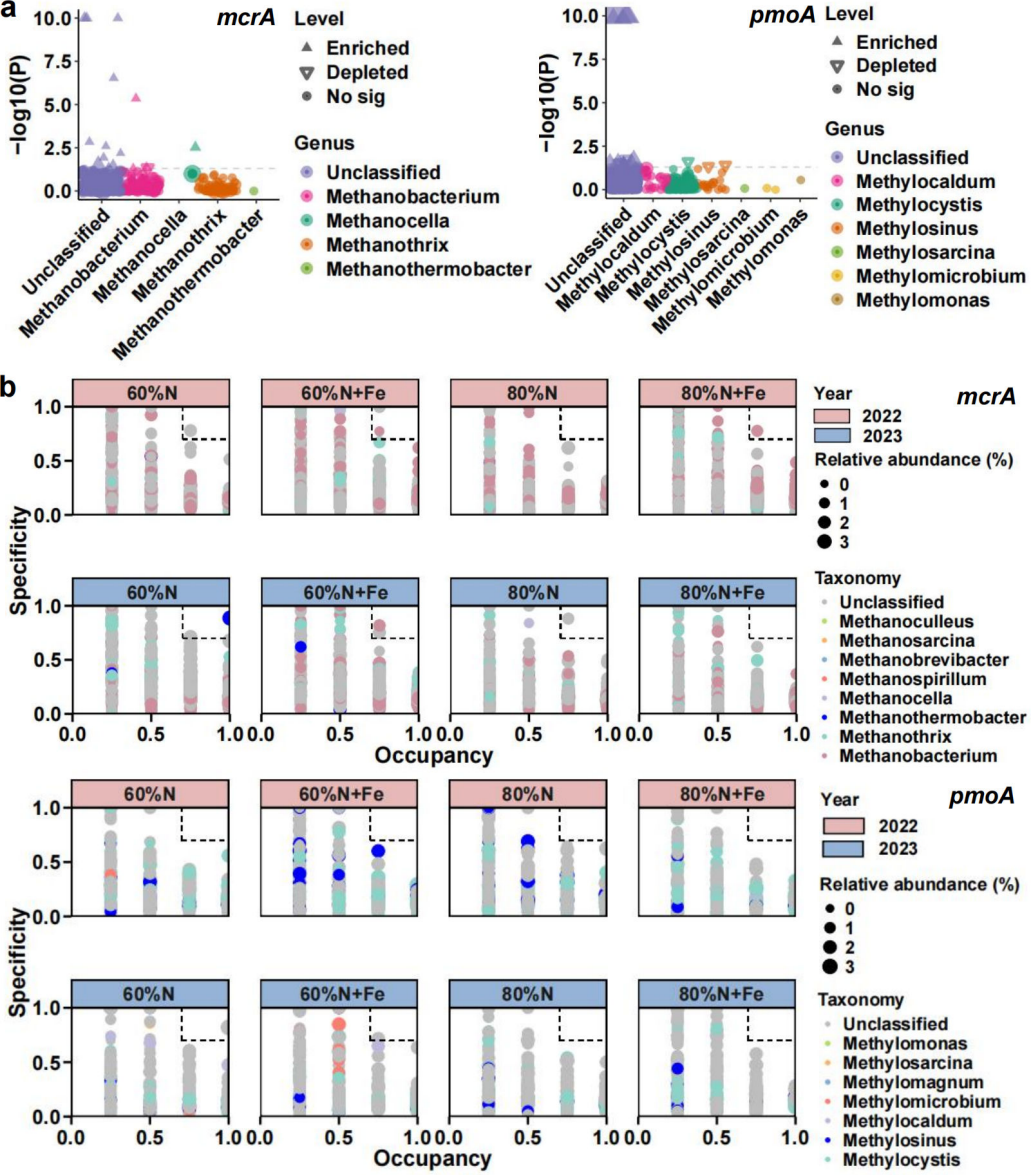
The Manhattan plot of the differential abundance analysis of methanogen and methanotroph communities (**a**). The specialists in methanogen and methanotroph communities (**b**).

In the methanogen community, a total of 16 differential species were screened out (*P* < 0.05). The Fe amendment significantly upregulated the abundances of 15 ASVs and significantly downregulated the abundance of 1 ASV (Fig. 5a). In the methanotroph community, 16 ASVs with differential abundance in different treatments were also observed (*P* < 0.05). The Fe amendment significantly upregulated the abundances of nine ASVs and significantly downregulated the abundances of seven ASVs (Fig. 5a). In the methanogen community, there were a total of 10 specialists, among which the 60%N + Fe treatment had the largest number of specialists (*n* = 5) (Fig. 5b). In the methanotroph community, two specialists were observed in total, with one specialist in the 60%N treatment and one in the 60%N + Fe treatment, respectively (Fig. 5b).

**Fig 6:**
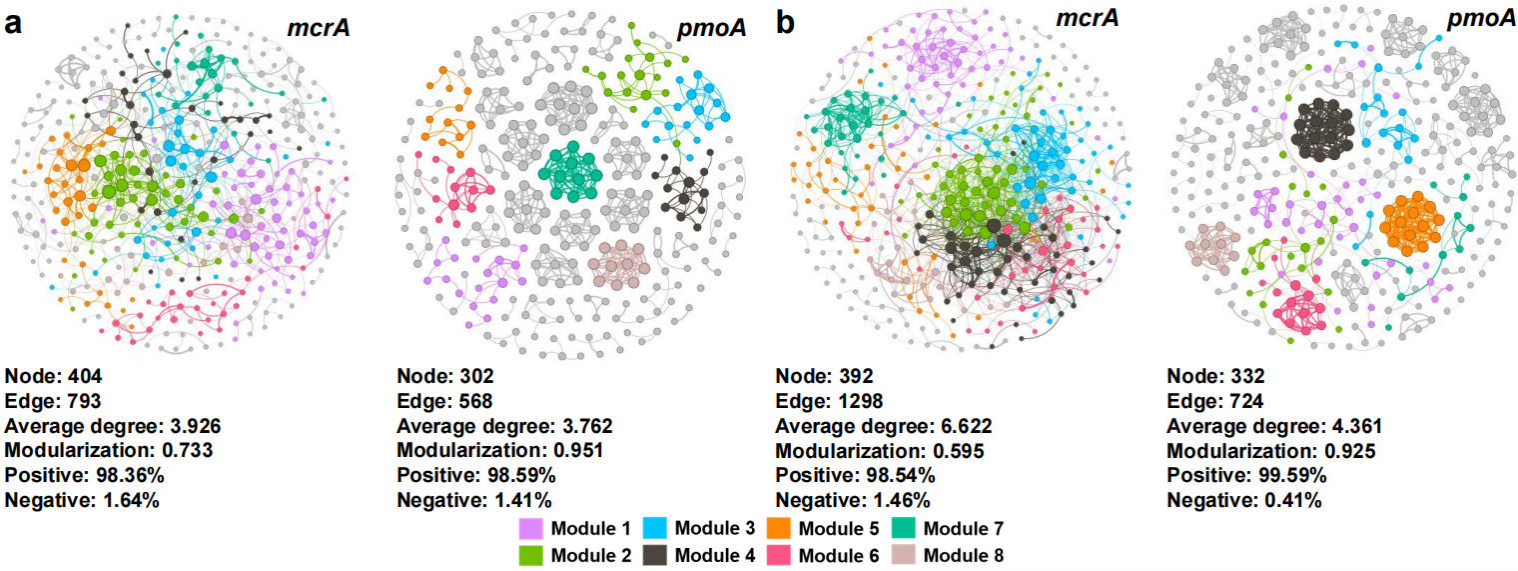
The co-occurrence networks of the methanogen and methanotroph communities in 2022 (**a**) and 2023 (**b**) rice seasons.

The co-occurrence network of the methanogen community had more nodes, edges, and a higher average degree compared to that of the methanotroph community, while the modularity degree was lower (Fig. 6a and b). In all co-occurrence networks, the correlations between ASVs were mainly positive correlations. For both the methanogen community and the methanotroph community, Fe amendment resulted in co-occurrence networks with more edges, a higher average degree, and lower modularity than the co-occurrence network without Fe amendment (Fig. 6a and b). The relative abundance of module 5 in the methanogen community was positively correlated to the cumulative CH_4_ emission (*P* < 0.01), while the relative abundance of module 2 in the methanotroph community was negatively correlated to the cumulative CH_4_ emission (*P* < 0.05, Fig. S5).

### Key factors driving methanogen and methanotroph community composition, diversity, and CH_4_ emissions

**Fig 7:**
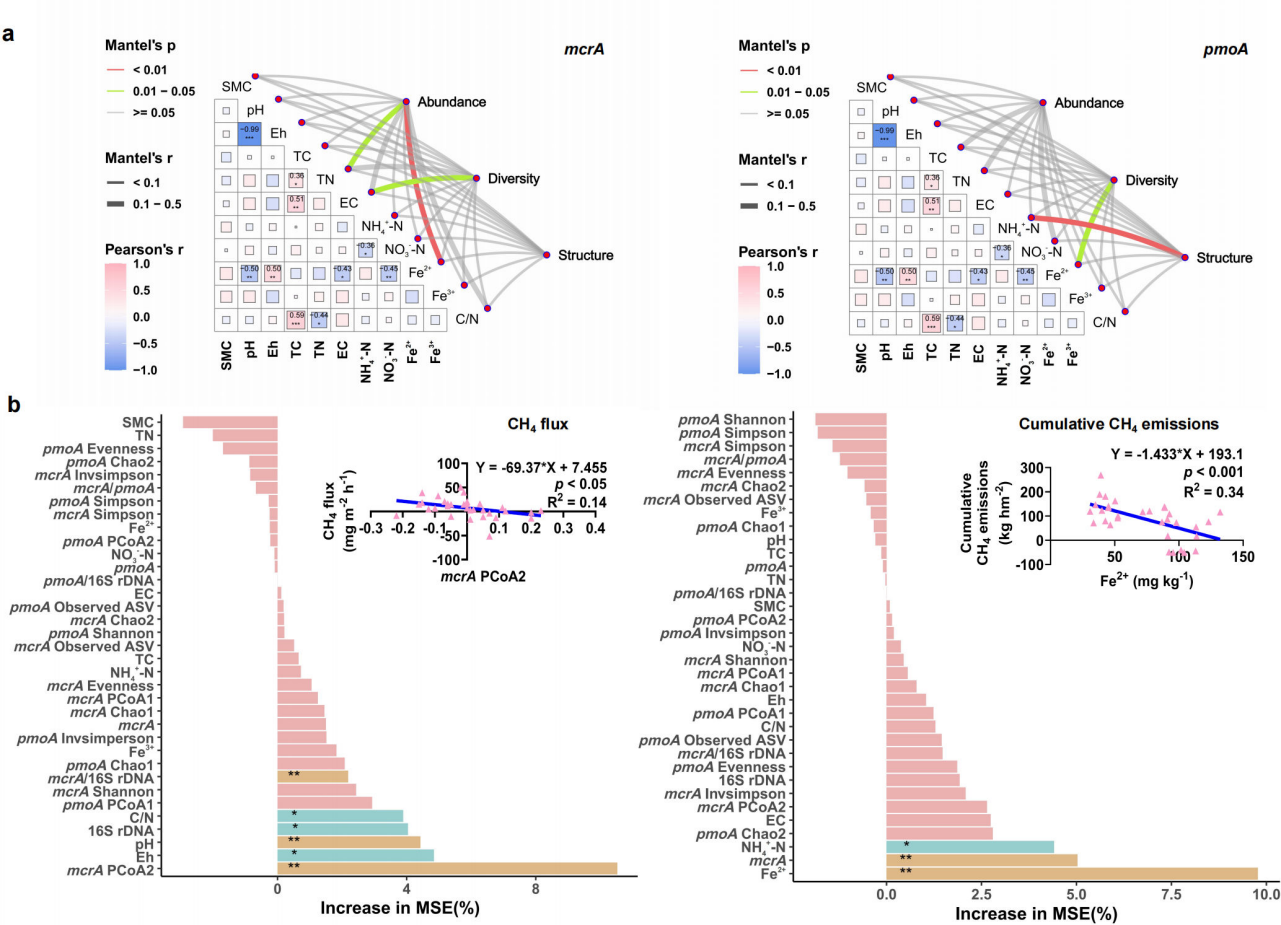
The correlation between soil physicochemical properties and diversity, structure, and abundance (**a**). The relative importance of predictors for CH_4_ flux and cumulative CH_4_ emissions based on random forests (**b**).

According to the Mantel correlation heatmap (Fig. 7a), the key environmental factors influencing the abundance of methanogens in paddy soil were mainly TN (*R* = 0.218, *P* < 0.05) and Fe^2+^ contents (*R* = 0.400, *P* < 0.01). The key factor influencing the community diversity of methanogens in paddy soil was mainly EC (*R* = 0.153, *P* < 0.05). The key factor influencing the community diversity of methanotrophs in paddy soil was mainly Fe^2+^ content (*R* = 0.141, *P* < 0.05), while the key factor influencing the community structure of methanotrophs in paddy soil was mainly NH₄^+^–N content (*R* = 0.127, *P* < 0.01). The random forest model (Fig. 7b) indicated that *mcrA* PCoA2, Eh, pH, 16S rDNA, C/N ratio, and *mcrA*/16S rDNA significantly influenced the CH_4_ flux in paddy fields (*P* < 0.05), while Fe^2+^, *mcrA*, and NH₄^+^–N significantly influenced the cumulative CH_4_ emissions from paddy fields (*P* < 0.05). Regression analysis further demonstrated that *mcrA* PCoA2 and Fe^2+^ were significantly negatively correlated with CH_4_ flux and cumulative CH_4_ emissions, respectively (*P* < 0.05, Fig. 7b).

## DISCUSSION

### Effects of reducing N fertilization rate and amendment with Fe on CH_4_ emissions, methanogens, and methanotrophs

Fe amendment rather than reducing N fertilization rate significantly decreased CH_4_ emissions (Fig. 1b and c), highlighting the complex interplay between N management and methanogenesis as well as the important inhibitory effect of Fe on CH_4_ emissions in subtropical paddies. CH_4_ emissions generally increase under flooded conditions like paddy fields (31), and the dynamic changes in flux in this study (Fig. 1a) also confirm this finding. Previous studies have shown that the application of a single Fe oxide can reduce CH_4_ emissions from rice paddies to a certain extent (32–34), while this study further found that under long-term N fertilizer reduction conditions, the application of Fe powder significantly decreased CH_4_ emissions from rice paddies (Fig. 1b and c). Actually, reducing synthetic N fertilizer application combined with Fe powder can also reduce the critical N losses in rice fields, enhance biological N fixation, and maintain rice yield (19–21). This study further illustrated the positive effects of reducing N fertilizer application combined with Fe powder in reducing environmental negative effects, which provides a novel solution for green and low N production in rice. However, it is worth noting that the Fe plaque formed around rice roots may significantly increase CH_4_ emissions from paddy soil by promoting the extracellular electron transfer between the methanogenic archaea and their syntrophic bacteria (35). Hence, it is still necessary to further investigate the differences in the effects of rhizosphere Fe and bulk soil Fe on CH_4_ emissions.

Although the response of methanogen and methanotroph community diversity to changes in fertilization patterns was limited, this study found that Fe application reduced the ratio of methanogen to methanotroph abundance compared to that without Fe amendment (Fig. 2). Fe amendments can inhibit soil organic matter decomposition, mainly by reducing the activity of methanogens and promoting CH_4_ oxidation processes to decrease CH_4_ emissions (26, 32, 36). When soil Fe reduction rates increase, CH_4_ production is simultaneously suppressed (37–39). Soil-derived dissimilative metal-reducing bacteria promote anaerobic methanogenesis through Fe reduction, competing with methanogens for substrates and decreasing their abundance and activity, thereby inhibiting CH_4_ generation (32, 36, 40). Generally, CH_4_ emissions are positively correlated with the *mcrA*/*pmoA* ratio (41, 42), which indicates that the dynamic balance between methanogens and methanotrophs has an important impact on CH_4_ emissions from paddy fields. In addition, previous studies only focused on community composition and diversity (32, 40); this study found that the formation mechanism of their community diversity varied under different N fertilizer rates with Fe amendment (Fig. 4). One important reason is that Fe can act as a nutrient and participate in metabolic processes, altering the competitive relationships and environmental adaptation strategies among microorganisms (43–46). Under the lower N rate (60%N), the addition of Fe may weaken the influence of deterministic factors such as species characteristics, interspecies interactions, and environmental conditions on the structure of methanogen community, but may enhance the influence of these factors on the methanotrophs. Notably, the application of Fe under 80% conventional N fertilization rate enhanced the stochasticity of the methanotroph community (Fig. 4b), which suggests the complexity of the methanotroph community in response to different fertilization patterns.

### Key taxa and soil physicochemical properties driving CH_4_ emissions

This study identified soil Fe^2+^ content as the key environmental factor driving cumulative CH_4_ emissions (Fig. 7b). Under anaerobic conditions, Fe^3+^ reduction outpaces methanogenesis (47). The Fe reduction process competes with the methanogenesis for electrons, which inhibits the growth of methanogens (32, 36, 40). Meanwhile, high concentrations of Fe^2+^ inhibit Fe^3+^ reduction, leading to a decrease in dissolved organic carbon (DOC) content released from Fe-bound organic carbon (48, 49). This decrease in DOC availability further inhibits the growth of methanogens, thereby suppressing CH_4_ emissions (48). This study further revealed that when soil Fe^2+^ content was high, the diversity of methanotroph communities also increased (Fig. 7a), which may be another indirect factor influencing CH_4_ emissions.

Key taxa may directly change the CH_4_ flux by regulating metabolic pathways, such as influencing the methanogenic pathway or the efficiency of CH_4_ oxidation (42). As the key methanogenic taxa affecting CH_4_ emissions between the Fe-amended and non-amended treatments, the ASV2385, ASV2330, and ASV2856 all belong to Methanobacteriota at the phylum level, with ASV2330 belonging to *Methanobacterium* at the genus level as well (Fig. S6). Previous studies have shown that Fe is a critical limiting factor for *Methanobacterium* growth, directly affecting CH_4_ production (50, 51). Additionally, these microorganisms may also have the ability to fix N_2_ (52). Hence, they were likely to have a key impact on CH_4_ emissions as well as the N cycle. Specialists have a lower tolerance to environmental fluctuations and are more affected by the environment (53). The relative abundance of the specialist ASV273, which was the most important taxon in the 60%N treatment, was closely related to the cumulative CH_4_ emissions (Fig. S6). This may also be the important reason for the higher CH_4_ emissions in the 60%N treatment compared to the 60%N + Fe treatment in this study (Fig. 1b and c). The amendment with Fe can reduce the complexity of the communities of methanogens and methanotrophs (26), while the complexity of the co-occurrence network in the Fe-amended treatment was generally higher than that in the non-amended treatment in this study (Fig. 6a and b). Actually, according to the neutral theory model (Fig. 4), as the deterministic processes weakened, interspecies competition may also weaken, leading to an increase in co-occurrence relationships between microorganisms and enhancing network connectivity. Interestingly, partial key modules were closely related to CH_4_ emissions only in non-amended treatments, while the role of modules in the network was weakened with Fe amendment (Fig. S5). The Fe amendment also weakened the modularity of methanogen and methanotroph communities (Fig. 6a and b). These shifts may indicate that the role of individual species was enhanced in the co-occurrence network (54). Recently, a study showed that changes in the community structure of methanogens and methanotrophs increased CH_4_ emissions from paddy fields in the climate change scenarios (55). This study clarified the importance of methanogen community structure in regulating CH_4_ flux (Fig. 7b). Moreover, the abundance of *mcrA* was also closely related to the cumulative CH_4_ emissions from subtropical paddy fields. This study indicated that the amendment with Fe powder combined with reducing synthetic N fertilizer, which has been a national strategic goal of “the Action Plan for Zero Growth in Fertilizer Use” in China, mainly altered the methanogen community rather than the methanotroph community, thereby playing a positive role in mitigating CH_4_ emissions from subtropical paddy fields.

### Conclusions

The 4-year field study demonstrated that Fe amendment combined with reduced N fertilizer application can significantly decrease CH_4_ emissions by approximately 57% in subtropical paddy fields, while saving up to 40% of synthetic N fertilizer use and maintaining rice yield. The Fe amendment significantly lowered the *mcrA*/*pmoA* ratio. The Fe amendment enhanced stochastic processes in the methanogen community at the 60% of conventional N rate, but reduced dispersal at the 80% of conventional N rate, with opposite trends observed for the methanotroph community. Co-occurrence networks of both methanogens and methanotrophs showed increased connectivity and reduced modularity with the Fe amendment. Soil Fe²^+^ content and methanogen community structure, as critical drivers, were negatively correlated to CH_4_ flux and cumulative emissions, respectively. Overall, reducing synthetic N fertilization combined with the Fe amendment offered a novel approach to mitigate CH_4_ emissions in subtropical paddy systems, contributing to climate-smart agriculture.

## SUPPLEMENTAL MATERIAL

Table S1 and Figures S1 to S6 spectrum.04000-25-s0001.pdf Results related to microbial abundance, community diversity, and key species.

ASM does not own the copyrights to Supplemental Material that may be linked to, or accessed through, an article. The authors have granted ASM a non-exclusive, world-wide license to publish the Supplemental Material files. Please contact the corresponding author directly for reuse.
