# Supplementary material for: Iron amendment decreases methane emissions from subtropical paddies by altering soil microbial communities
Source: Microbiol Spectr. 2026 Feb 24;14(4):e04000-25. doi: 10.1128/spectrum.04000-25 (PMC13055290; doi:10.1128/spectrum.04000-25)

Supplementary Material

Table S1 Real-time fluorescence quantitative PCR amplification primers

Gene	Primer	Primer sequences	qPCR reaction procedure
16S rDNA	341F	CCTACGGGNGGCWGCAG	95℃, 1 min; 95℃, 5 s; 55℃, 30 s; 72℃, 45 s; 39×
	806R	GGACTACHVGGGTATCTAAT	
mcrA	mlas-mod-F	GGYGGTGTMGGDTTCACMCARTA	95℃, 1 min; 95℃, 5 s; 55℃, 30 s; 72℃, 45 s; 39×
	mcrA-rev-R	CGTTCATBGCGTAGTTVGGRTAGT	
pmoA	A189F	GGNGACTGGGACTTCTGG	95℃, 1 min; 95℃, 5 s; 55℃, 30 s; 72℃, 45 s; 39×
	mb661R	CCGGMGCAACGTCYTTACC	

Fig. S1 Soil temperature and humidity in rice paddy fields

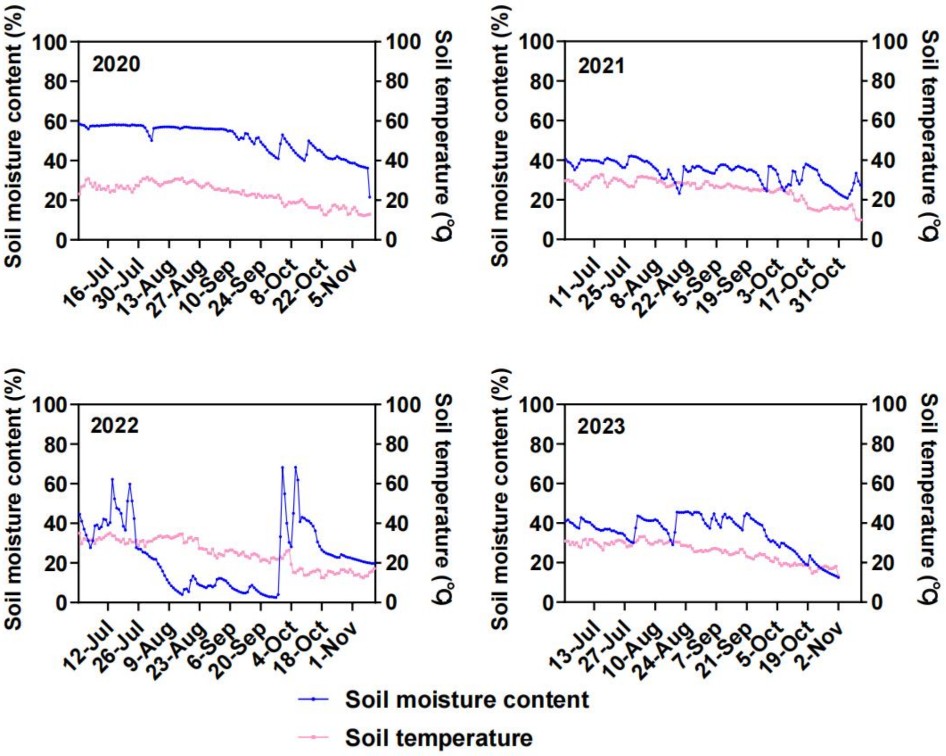

Fig. S2 Absolute and relative abundance of *mcrA* gene. 100%N, 80%N, 60%N and 0%N represented 100%, 80%, 60% and 0% conventional N fertilization rate, and 80%N + Fe, 60%N + Fe, 0%N + Fe represented 80%, 60%, 0% conventional N fertilization rate combined with Fe amendment, respectively. Statistical analysis was conducted using one-way and two-way ANOVA. Significant differences between treatments were tested by Tukey's HSD. Significance levels are indicated by N.S., not significant; *, $p < 0.05$; **, $p < 0.01$; ***, $p < 0.001$

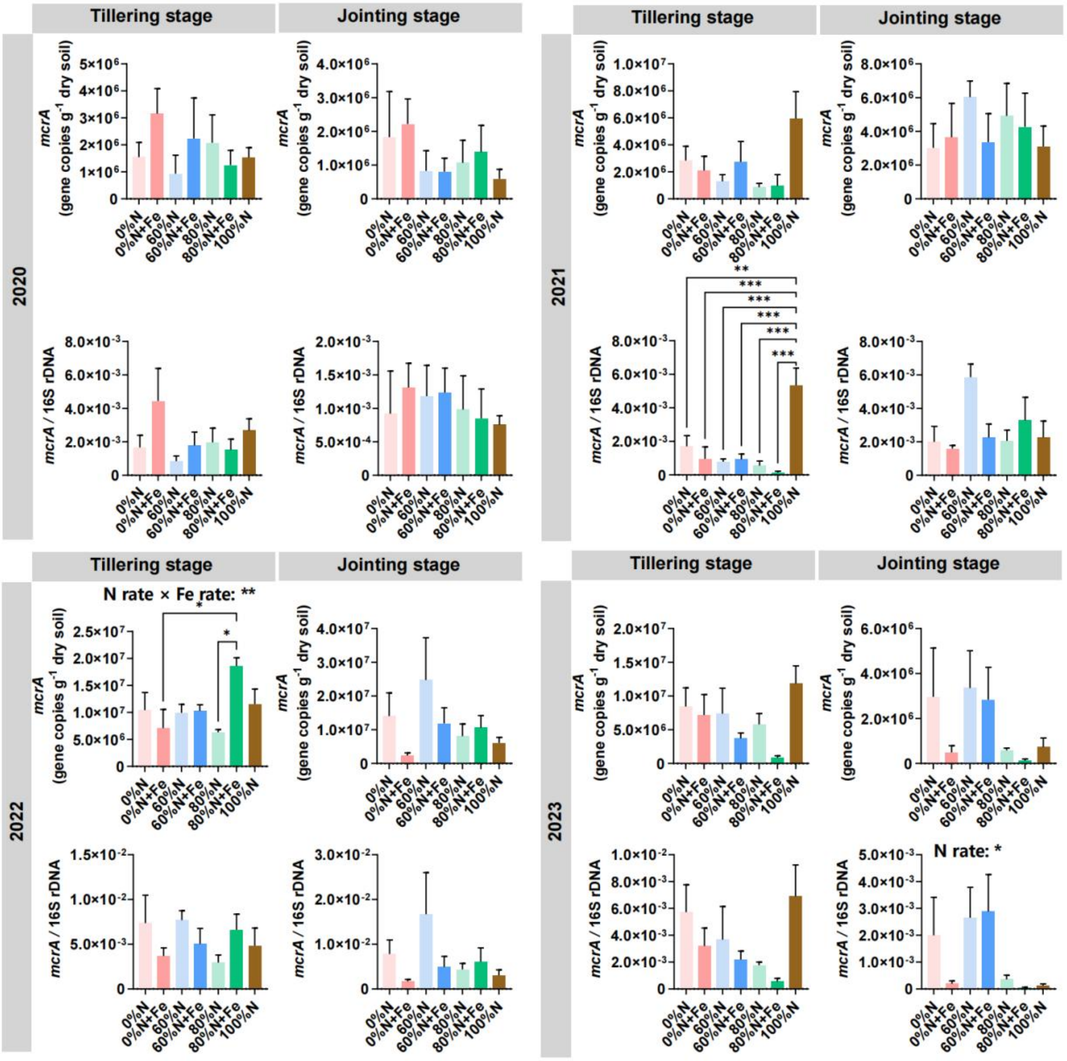

Fig. S3 Absolute and relative abundance of *pmoA* gene. 100%N, 80%N, 60%N and 0%N represented 100%, 80%, 60% and 0% conventional N fertilization rate, and 80%N + Fe, 60%N + Fe, 0%N + Fe represented 80%, 60%, 0% conventional N fertilization rate combined with Fe amendment, respectively. Statistical analysis was conducted using one-way and two-way ANOVA. Significant differences between treatments were tested by Tukey's HSD. Significance levels are indicated by N.S., not significant; *, $p < 0.05$; **, $p < 0.01$; ***, $p < 0.001$

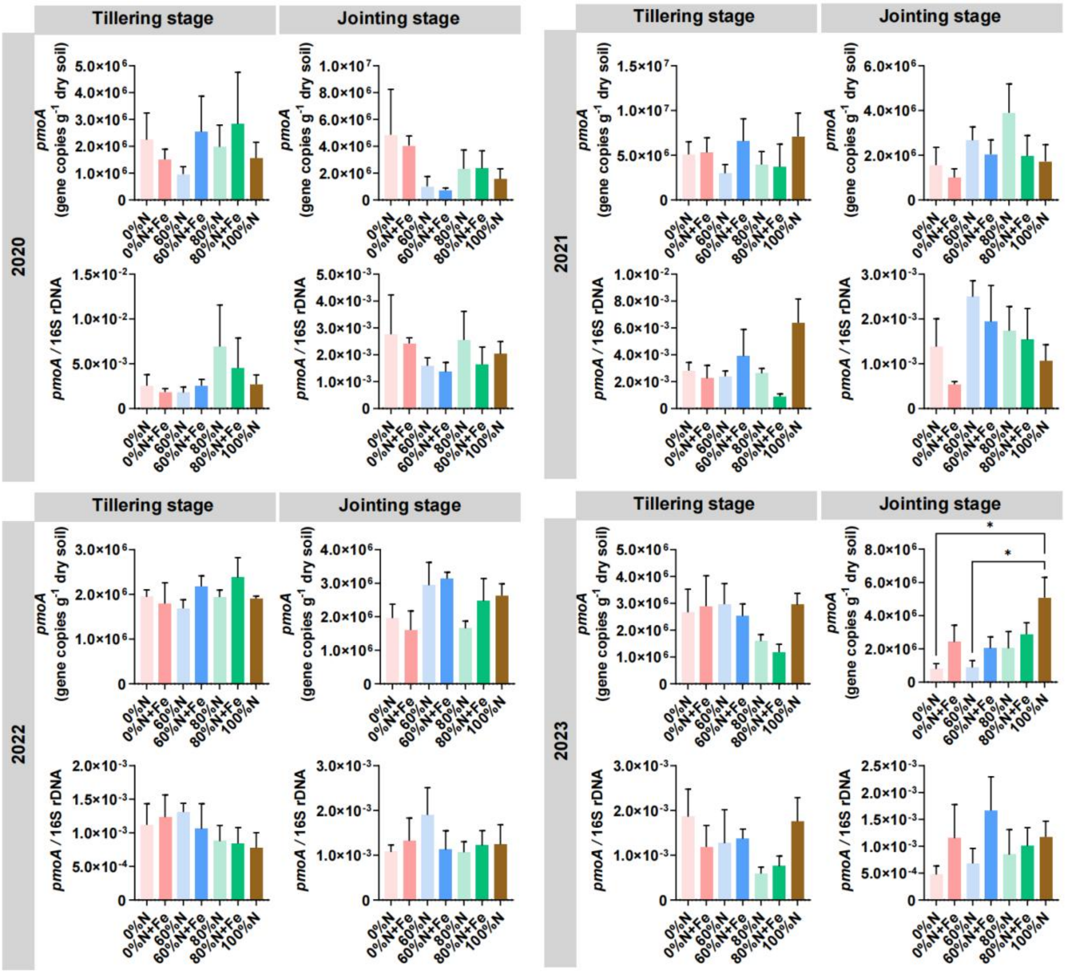

Fig. S4 Alpha and beta diversity of methanogenic communities (a) and methanotrophic communities (b). 80%N, 60%N represented 80%, 60% conventional N fertilization rate, and 80%N + Fe, 60%N + Fe represented 80%, 60% conventional N fertilization rate combined with Fe amendment, respectively. Statistical analysis was conducted using one-way ANOVA. Significant differences between treatments were tested by Tukey's HSD. The different letters above the plot boxes indicated the significance of the difference between different treatments.

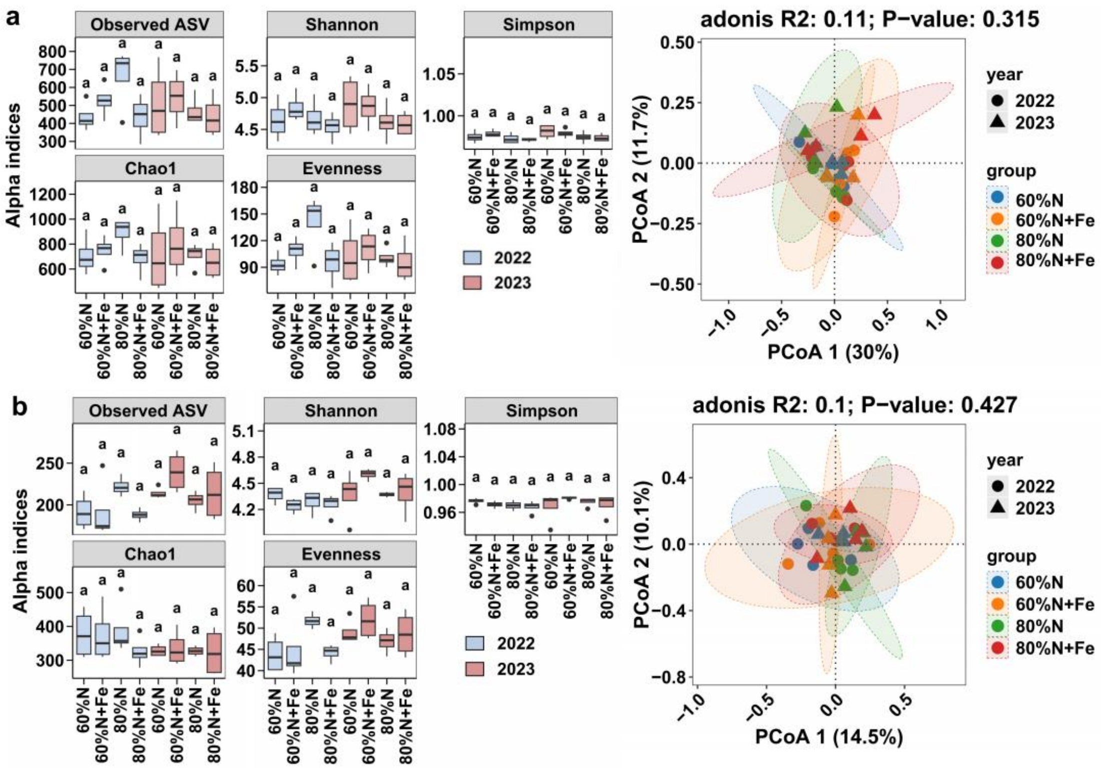

Fig. S5 The correlation heatmaps between the key modules of the methanogen community and soil physicochemical properties, CH_4 emissions (c), and the correlation heatmaps between the key modules of the methanotroph community and soil physicochemical properties, CH_4 emissions (d). Significance levels are indicated by N.S., not significant; *, $p < 0.05$; **, $p < 0.01$; ***, $p < 0.001$

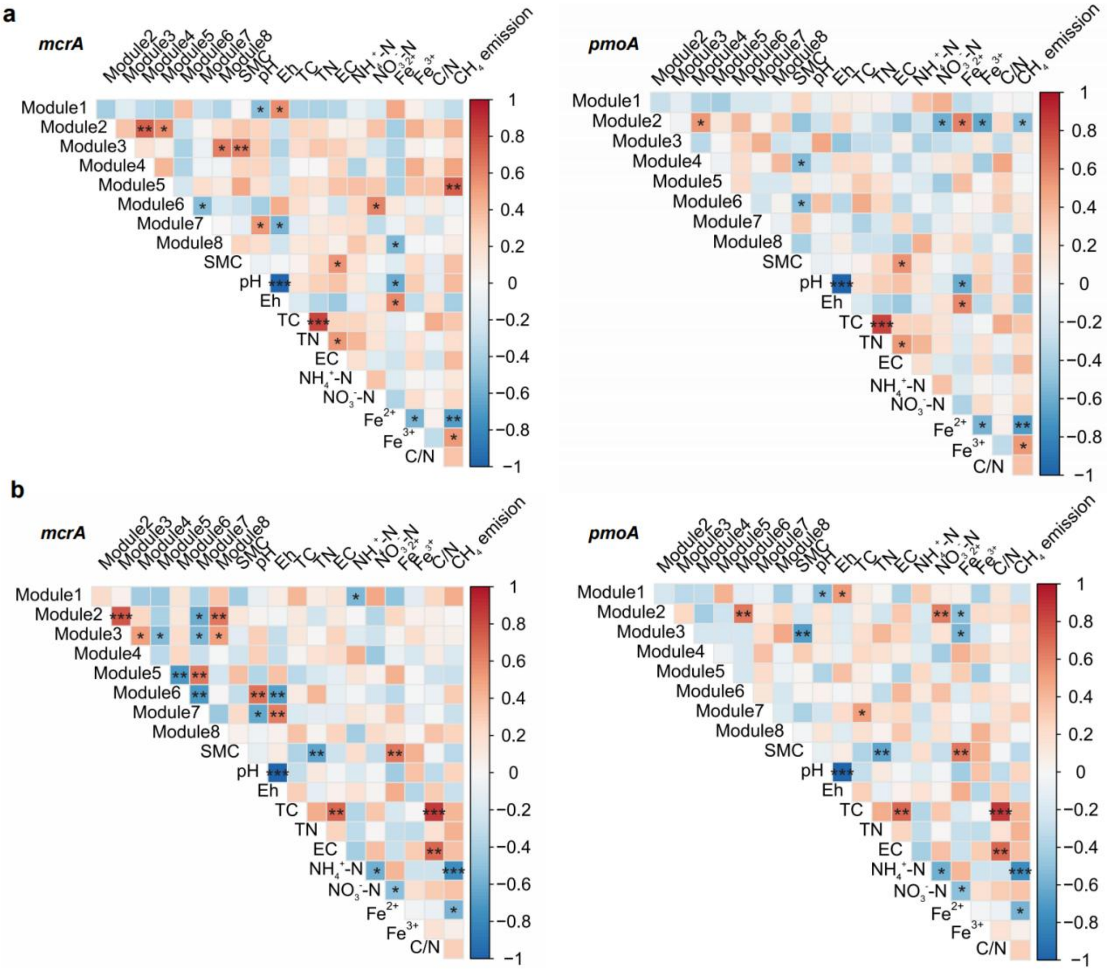

Fig. S6 Relative importance of differential abundance taxa and specialists in methanogenic and methanotrophic communities for CH_4 emissions. Significance levels are indicated by N.S., not significant; *, $p < 0.05$; **, $p < 0.01$; ***, $p < 0.001$

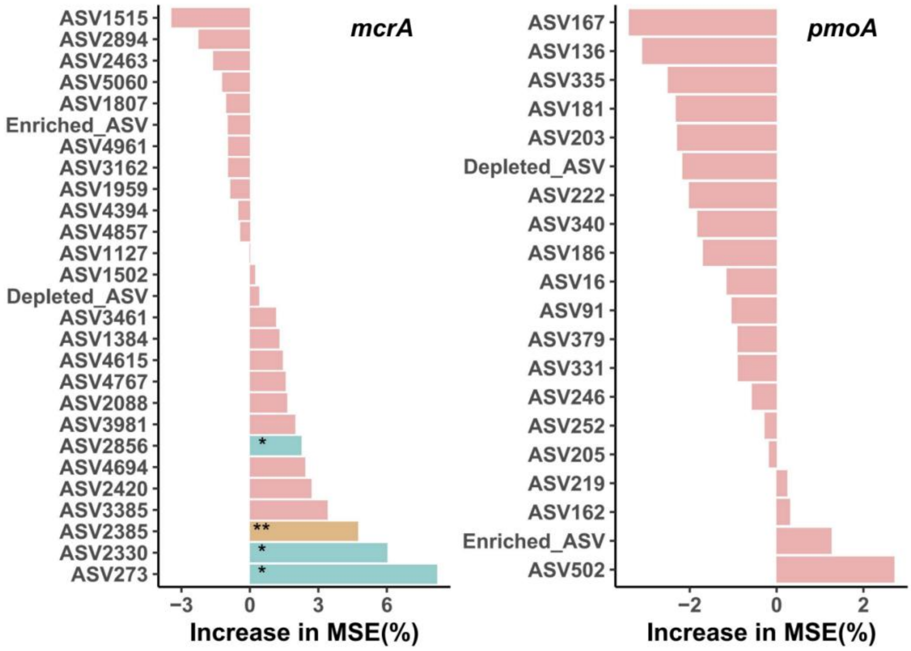

Supplement: Table S1 and Figures S1 to S6 — Results related to microbial abundance, community diversity, and key species. [file spectrum.04000-25-s0001.pdf]
